# Deceleration specific isometric exercises acutely enhance change of direction performance in male youth academy soccer players

**DOI:** 10.5114/biolsport.2026.159569

**Published:** 2026-04-20

**Authors:** Julián E. Smoliga, Viktor Oliva, Adrián Novosád, Matej Suja, Damian J. Harper

**Affiliations:** 1 Department of Track and Field and Strength & Conditioning, Faculty of Physical Education and Sport, Comenius University in Bratislava, Slovakia; 2 Department of Biological and Medical Sciences, Faculty of Physical Education and Sport, Comenius University in Bratislava, Slovakia; 3 Institute of Coaching and Performance, School of Health, Social Work and Sport, University of Central Lancashire, Preston, UK

**Keywords:** Isometric strength, Deceleration, Braking, Speed, Agility, Warm-up

## Abstract

Post-activation performance enhancement (PAPE) is a neuromuscular mechanism that can acutely enhance explosive movements such as change of directions (COD). The effectiveness of different deceleration specific isometric positions to elicit a PAPE effect on COD performance has not been previously investigated. Therefore, the aim of this study was to investigate the acute effects of two deceleration specific isometric exercises associated with the early (DEC_EISO_) and late (DEC_LISO_) deceleration subphases on COD performance. Sixteen male youth academy soccer players competing in the U16–U17 age categories completed both deceleration specific isometric protocols in a counterbalanced repeated-measures design. Performance was measured using the traditional 5-0-5 COD test at four intervals (0, 4, 8, and 12 minutes post-activation) for both right and left leg turns. Data were analysed using repeated measures ANOVA, with Bonferroni-adjusted post hoc tests. Parametric paired t-tests was used to compare differences in improvements between DEC_EISO_ and DEC_LISO_. Significant improvements in COD performance were observed following both deceleration specific isometric protocols, with the most pronounced gains occurring at the 8^th^ minute. At the 8^th^ minute, performance was significantly better following the DEC_EISO_ compared to the DEC_LISO_ (p = 0.002), with a large effect size (g = 0.90). These findings suggest that deceleration specific isometric exercise can have a significant impact on enhancing short-term COD performance. The DEC_EISO_ position produced superior results, particularly in the 8^th^ minute postactivation, highlighting its potential usefulness in pre-training or competition warm-ups for COD-based sports like soccer.

## INTRODUCTION

Post-activation performance enhancement (PAPE) refers to the acute increase of explosive neuromuscular capacity [1]. This effect can be induced by conditioning activities (CA) performed at maximal or near-maximal intensity [1, 2]. These CA are commonly used in warm-ups or integrated into training programmes to enhance strength, speed, and power outputs through neural activation, motor unit recruitment and physiological changes, such as increased muscle temperature and blood flow [1, 3]. The PAPE response, however, results from a balance between potentiation and fatigue, which develop simultaneously following a CA [4]. If fatigue outweighs potentiation, performance may not improve. The outcome depends on several modulating factors, including training status, CA type and intensity, recovery interval and biomechanical specificity [1, 4, 5].

In team sports such as soccer, COD ability, requiring rapid accelerations and decelerations, is critical for player performance and match outcomes [6, 7]. Consequently, those responsible for preparing soccer players are very interested in training approaches that could enhance players COD performance. The use of PAPE to provide acute COD performance enhancements has been studied extensively, with a systematic review and meta-analysis of 34 studies (19 in meta-analysis) reporting that a range of CA (i.e., unloaded, light, moderate-to-heavy loaded) can significantly improve COD performance compared to control conditions in male athletes (SMD = .49), with unloaded and light loaded (0–30% body mass) CA resulting in the greatest performance effects [8]. Despite these findings, the review did not consider potential modulating factors related to the resistance training modality (i.e., isoinertial, isoweight, isokinetic), training method (isometric, eccentric, coupled concentric-eccentric) or direction of load (i.e., horizontally or vertically orientated exercises).

For sharp COD movements (i.e., angles > 70°) that requires generation of very high forces and orientation of forces in the horizontal direction, PAPE exercises that emphasise high forces particularly with a horizontal focus may offer superior PAPE effects. The research in this area incorporating male soccer players, however, remains controversial. Petisco et al. [9] reported that moderate intensity half squats (80% of 1RM) acutely enhanced COD performance (T-Test) compared with low (60% of 1RM) or maximal (100% of 1RM) intensities in professional soccer players. In contrast, Aytac et al. [10] reported that heavy back squats (90% 1RM) did not enhance COD performance (505 and T-Test) at any measured time point (i.e., 15 s, 2 min, 4 min, 8 min, 12 min and 5 min post-CA) in strength trained team sport athletes that included soccer players. Beyond traditional dynamic resistance training approaches, flywheel eccentric exercises have been used as an alternative strategy to induce PAPE effects on COD performance. Beato et al. [11] reported that a half-squats with medium to high inertial loads enhanced COD after 3–6 minutes in physically active males including soccer players. To examine differences between exercise types, Beato et al. [12] compared horizontal (i.e., cross-cut step) and vertical (i.e., unilateral squat, leg extension) force vector exercises. All flywheel exercises enhanced COD performance after 4 minutes, likely due to improvements in muscle contractile function (i.e., increased stiffness and enhanced muscle reactivity to signal transmission). In contrast, McErlain-Naylor et al. [13] reported no PAPE effects on horizontal ground reaction forces during a modified 5-0-5 COD after a flywheel squat, suggesting PAPE effects could be more pronounced using horizontally focused exercises. This could be particularly plausible in COD tests that demand significant deceleration (i.e., 180-degree COD) prior to re-accelerating, such as in the traditional 5-0-5 COD test or modified versions of it.

While flywheel isoinertial methods offer dynamic overload and benefits associated with eccentric muscle strength and braking capabilities, isometric exercises provide a contrasting yet promising alternative. By emphasizing maximal force production without joint movement, isometric CA may stimulate enhancements in neuromuscular function (i.e., muscle activation, rate of force development) with relatively low fatigue [14] that could be particularly beneficial for enhancing rapid deceleration and sharp COD movements. Furthermore, isometric exercises can be used to target joint specific strength improvements observed when performing sport specific movements such as decelerations and COD [14]. Evidence on use of isometric CA for enhancing COD performance is limited [15–18]. While some studies found no effect [17], others reported significant improvements [18]. Marshall et al. [17] used 3 × 3 s isometric half squats with a knee angle of 90° and observed no enhancement in COD performance (5-10-5 test) after rest periods of 1, 3, 5 and 7 minutes. Using the same isometric protocol with youth soccer players, Toprak et al. [18] reported no changes in COD performance (Illinois test) compared to a control warm-up following rest periods of 15 s and 2 mins, but significant improvements were observed between 4 and 14 mins. These findings suggest that isometric PAPE protocols could acutely enhance COD performance in youth soccer players when appropriately timed, but that further research is needed to investigate this in youth soccer players.

In sharp COD tasks, the deceleration phase requires generation of very high forces to rapidly reduce momentum prior to turning and is therefore integral to attaining faster COD performance [19]. This, therefore, makes it an ideal target for application of CA for PAPE of sharp COD actions. The deceleration phase can be divided into early and late deceleration sub-phases, with braking steps in the early deceleration phase having a more upright posture and less upright posture for lower limb joint flexion throughout stance compared to braking steps in the late deceleration phase. Steps in each phase may contribute differentially to enhanced COD performance. For example, early braking steps are key for quickly reducing momentum, which may contribute to less braking demands in the late deceleration phase, whereas braking steps in the late deceleration phase may help to enhance whole body positioning prior to turning and re-accelerating. Currently, however, no study to the author’s knowledge has investigated the effects of isometric exercises targeting braking specific positions observed in the early and late deceleration subphases on COD performance. Therefore, the aim of this study was to investigate the acute effects of two deceleration specific isometric exercises on COD performance. We hypothesised that both protocols would significantly improve COD, with peak effects observed between 4–8 minutes post-activation.

## MATERIALS AND METHODS

### Experimental Approach to the Problem

A counterbalanced repeated-measures study design was used to determine PAPE effects of two different deceleration specific isometric positions on COD performance. The two-deceleration specific isometric positions were adapted from a deceleration specific isometric exercise previously described by Harper et al. [14] associated with the early (DEC_EISO_) and late deceleration (DEC_LISO_) subphases that occur prior to any sharp COD (i.e., > 90 degrees). Following performance of each deceleration specific isometric exercise, the 5-0-5 COD test was performed and then repeated at 4, 8 and 12 minutes to determine the most optimal time period for PAPE.

### Subjects

Sixteen male youth academy soccer players from U16 and U17 age category (mean ± *SD*; age = 16.17 ± 0.69; height = 176.7 ± 4.07 cm; body mass = 68.0 ± 4.03 kg) volunteered to participate in the study. All subjects were playing for the same academy, training together on daily basis and were familiar with performing regular COD movements such as the 5-0-5 COD test. To be eligible for inclusion all subjects were required to be without any injury or medical conditions in the last nine months and complete a 4-week familiarisation process that involved performing isometric training twice a week in DEC_EISO_ and DEC_LISO_ positions. Subjects were informed of the benefits and risk of the investigation prior to attaining voluntary informed written assent and parental consent due to age. The study was approved by the Ethics Committee of Faculty of Physical Education and Sport (REF: 21/2024).

### Procedures

#### Familiarisation Session

**FIG. 1:**
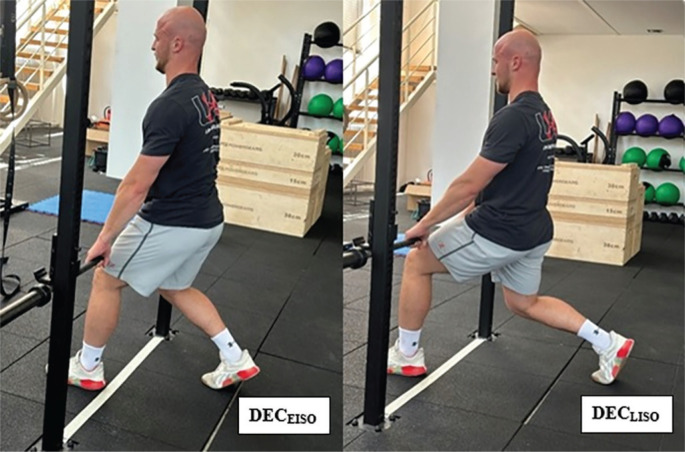
Deceleration specific isometric exercises associated with the early (DEC_EISO_) and late (DEC_LISO_) deceleration sub-phases.

The familiarization process lasted four weeks, with two training sessions per week, during which participants performed isometric training in deceleration specific push isometric positions. The training protocol consisted of 3 × 3-second deceleration specific push isometric exercise [17] per leg performed in the DEC_EISO_ and DEC_LISO_ positions (Figure 1). Each participant’s DEC_EISO_ and DEC_LISO_ positions were individually adjusted using a manual goniometer, ensuring a knee joint angle of 160° for DEC_EISO_ and 110° for DEC_LISO_ [14]. Participants were instructed to pull the bar and push through the legs as fast as possible for a duration of 3 seconds, with a three-second rest between repetitions and 10 seconds rest between legs. Each participant completed three sets per session with 90 seconds rest between sets.

**FIG. 2:**
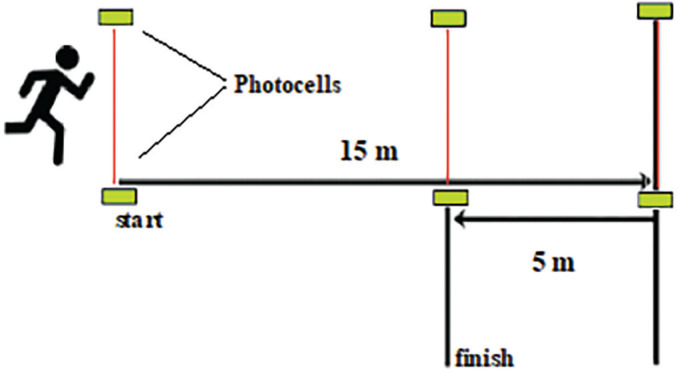
Set up of the 5-0-5 change of direction test.

Following the deceleration specific isometric exercises each participant performed the 5-0-5 COD test with a right leg turn and after 30 seconds rest repeated the test with a left leg turn. The 5-0-5 test has been described previously and used extensively for assessing COD in team sports [20–22]. For soccer players it has been recommended as a valid assessment of COD performance due to it mirroring the peak speeds attained when pressing during matches [20–22]. Participants started in a split-stance position at a designated starting line and sprinted 15 meters at maximal effort. Upon reaching the 15-meter mark, they performed a 180° turn before immediately re-accelerating into a 5-meter sprint in the opposite direction (Figure 2).

#### Testing Session

Testing took place 7 days after the familiarization process across a period of 4-weeks. To account for potential circadian variation, all testing took place each week on the same day and time. Firstly, all participants performed a 3-minute light jog at an intensity corresponding to RPE 4–5/10, during which they were able to comfortably maintain a conversation. This was followed by a standardised dynamic warm-up consisting of 13 exercises (knee hug, leg cradle, quadriceps stretch, reverse lunge to hamstring stretch, inchworm, spiderman stretch, lateral lunge, high knees, butt kicks, carioca, backpedal, A-skip, straight-leg march). Each exercise was performed between 10-meter lines interspersed with a short walk back to the start line [23]. Two minutes later, all participants performed a baseline measure of the 5-0-5 COD test from a right leg turn and a left leg turn following 30 seconds of recovery. The 5-0-5 COD test was measured by single-beam photocells (Witty, MicroGate NEXT, Italy). Photocells placed at the 10 m mark to record the COD performance time (i.e., 5 m before and after the turn). The reliability coefficient between baseline tests of DEC_EISO_ and DEC_LISO_ protocols for COD test was good (Intraclass correlation coefficient (ICC) for single measurements = 0.871; 95% confidence interval (CI) = 0.751–0.947). After 2 minutes of passive recovery participants performed 3 × 3 second of the deceleration specific isometric exercise with 3 seconds rest period between legs. The DEC_EISO_ and DEC_LISO_ positions for each subject was determined prior to the familiarization process. The selection of DEC_EISO_ and DEC_LISO_ was done randomly by throwing a coin by each participant after the baseline testing. Once completed participants were re-tested after 4, 8 and 12 minutes. Similar to baseline testing procedures, subjects always began the COD test with a right leg turn and after 30 seconds of recovery with a left leg turn. During testing, all participants were instructed not to use any active recovery techniques or perform preparatory sprints.

### Statistical Analyses

All statistical analysis was conducted using IBM SPSS Statistics version 27.0 (IBM Corp., New York, USA). To assess the normality of data distribution, the Shapiro-Wilk test was applied. A two-way and one-way repeated-measures ANOVA, followed by a Bonferroni post hoc test, was utilized to examine the potentiation effects of the DEC_EISO_ and DEC_LISO_ protocols on the dependent variable (COD time in seconds). The analysis considered four time points (0, 4, 8, and 12 minutes) and two protocols (DEC_EISO_ and DEC_LISO_). To assess differences between DEC_EISO_ and DEC_LISO_ parametric paired *t*-test with estimated Hedges’ *g* effect size were used. The magnitude of differences was interpreted using the following scale [24]: < 0.19 = trivial, 0.20 to 0.49 = small, 0.50 to 0.79 = medium and > 0.80 = large. To determine the reliability of the baseline data, the ICC with a 95% confidence interval was calculated across two testing sessions [25]. Specifically, the ICC was computed between the baseline values of the DEC_EISO_ and DEC_LISO_ protocols. Alongside ICC, the coefficient of variation (CV) was calculated to assess absolute reliability [26]. Additionally, the standard error of measurement (SEM) and the smallest real difference (SRD) were analysed following the approach by Beckerman et al. [27]. SEM was calculated using the formula: SEM = σ × √(1 − r) (where σ represents the standard deviation and r is the reliability coefficient), while SRD was determined as 1.96 × √2 × SEM, accounting for variance differences between conditions. SWC was calculated as 0.2 times the between-subject standard deviation of the baseline measurements (SD = 0.054). Practical significance was evaluated using Partial Eta Squared, specifically focusing on the time effect. The Partial Eta Squared values were interpreted based on the following scale: < 0.009 = negligible, 0.010 to 0.059 = small, 0.060 to 0.139 = moderate and > 0.140 = large [24]. The level of statistical significance was set at p < 0.01 and p < 0.05 with a 95% confidence interval. For descriptive statistics, means and standard deviations (SD) were reported.

## RESULTS

The ICC for average measures was excellent (0.953; 95% CI: 0.909 – 0.979; *p* < 0.001) while for single measures was good (0.871; 95% CI: 0.751 – 0.947; *p* < 0.001) with a CV of 1.37 %, confirming strong internal consistency and good to excellent measurement reliability. The SEM, SRD and SWC were 0.020 s, 0.054 s and 0.011 s, respectively.

A two-way repeated measures ANOVA (2 protocols × 4 time points) revealed a significant main effect of time on performance in the 5-0-5 COD test for both left and right leg turns. The quadratic trend was especially pronounced (right leg: *F*_(1,15)_ = 19.028, *p* < 0.001, η^2^ = 0.559; left leg: *F*_(1,15)_ = 25.386, *p* < 0.001, η^2^ = 0.629), indicating initial performance improvements followed by a plateau or decline. A significant cubic trend was also present, suggesting a more complex temporal pattern.

The main effect of protocol (DEC_EISO_ vs. DEC_LISO_ position) was not statistically significant (right leg: *p* = 0.984; left leg: *p* = 0.252). However, a significant protocol × time interaction was observed for the right leg in the cubic trend (*F*_(1,15)_ = 4.585, *p* = 0.049, η^2^ = 0.234), indicating that the performance trajectory over time differed between protocols.

**TABLE 1:** Comparison of differences in 5-0-5 change of direction (COD) performance (mean ± SD) between early (DEC_EISO_) and late deceleration (DEC_LISO_) specific isometric protocols across time points.

Deceleration Specific Isometric Protocol	COD (0^th^ minute)	COD (post 4 min)	COD (post 8 min)	COD (post 12 min)	Time curve	p value	partial η^2^
**DEC_EISO_ right leg**	4.084 ± 0.059 s	4.048 ± 0.065 s	4.004 ± 0.052 s **	4.064 ± 0.064 s	Linear Quadratic Cubic	0.066 **< 0.001** **0.006**	0.208 0.531 0.404

**DEC_EISO_ left leg**	4.080 ± 0.057 s	4.041 ± 0.083 s	3.997 ± 0.068 s **	4.062 ± 0.063 s	Linear Quadratic Cubic	**0.015** **0.005** **0.005**	0.337 0.426 0.413

**DEC_LISO_ right leg**	4.077 ± 0.049 s	4.044 ± 0.046 s *	4.027 ± 0.044 s **	4.053 ± 0.049 s	Linear Quadratic Cubic	0.097 **0.010** 0.411	0.173 0.370 0.045

**DEC_LISO_ left leg**	4.082 ± 0.056 s	4.059 ± 0.054 s	4.016 ± 0.074 s *	4.071 ± 0.069 s	Linear Quadratic Cubic	0.189 **0.002** **0.008**	0.112 0.471 0.384

Note:

* = < 0.05;

** = < 0.01

**FIG. 3:**
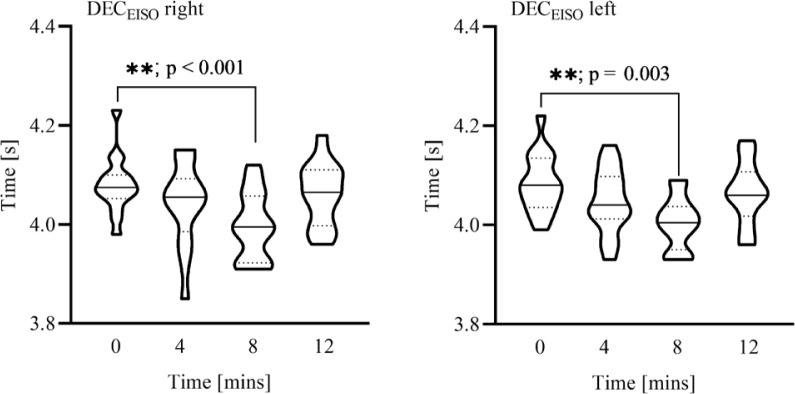
Difference in 5-0-5 change of direction performance using the early deceleration specific isometric protocol (DEC_EISO_).

In both protocols (DEC_EISO_ and DEC_LISO_), significant quadratic effects of time were found, indicating non-linear performance changes. For the DEC_EISO_ protocol, quadratic effect shown statistically significant trend for the right leg (*F* = 16.961, *p* < 0.001, η^2^ = 0.531) and for the left leg there was linear and also quadratic significant trend of performance (linear: *F* = 7.614, *p* = 0.015, η^2^ = 0.337; quadratic: *F* = 11.136, *p = 0.005*, η^2^ = 0.426). Bonferroni post hoc tests confirmed that the main performance improvement for the right and for the left leg occurred between the 0^th^ and 8^th^ minute (right leg: *p* < 0.001; left leg: *p* = 0.003) (Figure 3).

**FIG. 4:**
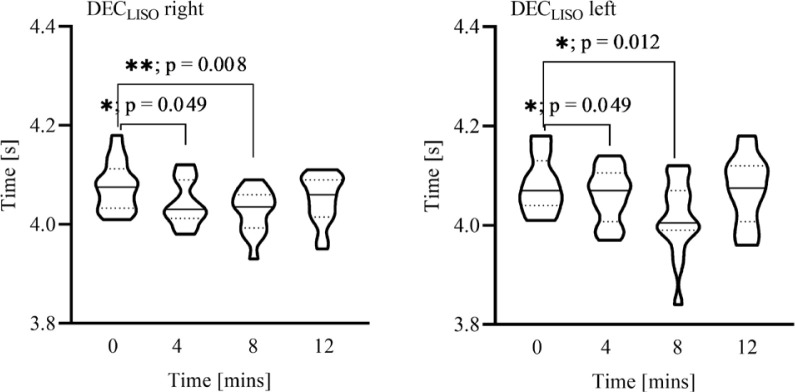
Difference in 5-0-5 change of direction performance using late deceleration specific isometric protocol (DEC_LISO_).

Similarly, the DEC_LISO_ protocol showed a significant but smaller quadratic effect for the right leg (*F* = 8.796, *p* = 0.010, η^2^ = 0.370) and significant quadratic effect with big practical effect for the left leg (*F* = 13.335, *p* = 0.002, η^2^ = 0.471) in changes of COD performance. The Bonferroni post hoc tests indicating significant improvement between the 0^th^ and 4^th^ minute (*p* = 0.043) and 8^th^ minute for the right leg (*p* = 0.008) and for the left leg between 0^th^ and 8^th^ minute only (*p* = 0.012). No other comparisons reached statistical significance in either protocol (*p* > 0.05) (Figure 4).

**FIG. 5:**
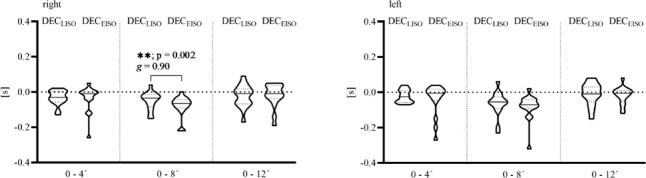
Differences between early (DEC_EISO_) and late deceleration (DEC_LISO_) specific isometric protocols on 5-0-5 change of direction performance for right and left leg turns.

Paired t-tests revealed that a significant COD performance difference between protocols (DEC_EISO_ vs DEC_LISO_) occurred only at the 8^th^ minute for the right leg (*t*(15) = 3.704, *p* = 0.002), in favour for the DEC_EISO_. The effect size was large (Hedges’ *g* = 0.90). No significant differences were observed at other time points or for the left leg (*p* > 0.05), with trivial effect sizes (Hedges’ *g* < 0.2) (Figure 5).

Following the DEC_EISO_, the observed COD performance improvement between 0 and 8 minutes was 0.079 ± 0.061 s for the right leg. This change exceeded both SWC (0.011 s) and SRD (0.054 s), indicating a meaningful and reliably measurable improvement. Conversely, the performance gain following the DEC_LISO_ protocol during the same interval (0.050 ± 0.051 s) exceeded the SWC but not the SRD, suggesting a change that is meaningful in training terms but possibly within the bounds of measurement error.

## DISCUSSION

The aim of this study was to investigate the acute PAPE effects of two deceleration specific isometric exercises (DEC_EISO_ and DEC_LISO_) on COD performance in youth academy soccer players. The key finding of this study was that both deceleration specific isometric protocols elicited significant improvements in COD performance, with the most pronounced effect occurring 8 minutes post-activation. Notably, the DEC_EISO_ resulted in significantly greater improvement in COD performance compared to the DEC_LISO_, highlighting this position to be particularly effective for obtaining acute enhancements in COD performance.

The finding that deceleration specific isometric exercises acutely enhanced COD performance is a novel finding. To the authors knowledge, no study has previously investigated the use of isometric exercises in deceleration specific positions aligned with the early and late deceleration sub-phases that occur prior to any sharp COD movement. Previous studies that have used isometric exercises to acutely enhance COD performance have used mostly traditional resistance training exercises, such as bilateral half back squats at different angles [15–18] and unilateral half back squats [15]. Two of these studies [15, 17] did not show significant effects on COD performance. In contrast, Toprak et al. [18] and Pojskić et al. [16] reported significant improvements in COD performance following isometric squats at 5 minutes post-CA. Recent designs combining isometric efforts with dynamic loading yielded variable results. For example, Toprak et al. [18] combined heavy squats (85% 1RM) with maximal isometric efforts and observed faster COD performance at 4–6 minutes, peaking at 8 minutes post-CA, while Kalinowski et al. [15] found no meaningful changes after pairing isometric half squats with bilateral drop jumps. In the present study, both DEC_EISO_ and DEC_LISO_ protocols enhanced COD performance in COD performance at 8 minutes on both limbs. These findings support previous research demonstrating that the timing and nature of CA can critically determine the presence and magnitude of a PAPE effect [28, 29].

Another interesting finding from the current study was that while both DEC_EISO_ and DEC_LISO_ protocols improved COD performance, the DEC_EISO_ protocol resulted in greater and more consistent gains comparing to DEC_LISO_ protocol. This finding [30] could be due to the critical role of the early deceleration subphase for overall deceleration performance [31] where the ability to generate higher horizontal braking forces at higher speeds would enable less distance and time to reduce momentum prior to directional change resulting in faster overall COD performance [31]. Furthermore, the highest magnitude of forces (~6 × body weight) occur during braking steps of the early deceleration sub-phase and can be up to 2.7 times greater than those seen during the first steps of a maximal horizontal acceleration [19]. Therefore, the ability to generate and tolerate these forces may further explain why DEC_EISO_ protocol may have significantly enhanced COD performance at 8^th^ minute when compared to DEC_LISO_ protocol. Whilst the DEC_LISO_ still produced significant enhancements in COD performance, it may not sufficiently replicate the key mechanical demands of the early deceleration sub-phase. Although some significant gains were observed, notably at the 8-minute period, they did not exceed the SRD, suggesting that such changes may fall within normal measurement variability and thus carry lower practical value. Therefore, based on these findings, it is recommended to use the DEC_EISO_ position to instigate acute PAPE effects on sharp COD performance that involves significant deceleration prior to turning.

Our study had some limitations. We did not measure the force production or neuromuscular outputs in either isometric protocol. Future research should therefore incorporate neuromuscular (e.g., muscle activation) or biomechanical (e.g., force plates) monitoring to further elucidate underlying mechanisms that may have enhanced deceleration and COD performance. Additionally, to better determine the influence of the isometric protocols on different sub-phases of COD (i.e., acceleration, deceleration and re-acceleration), equipment that can measure continuous velocity would give more in-depth insights compared to timing gates that are restricted to measures of total time. Future research should investigate the effects of deceleration-specific isometric protocols in other team sports, different standards of competition, age groups, and in female athletes to provide broader insight into the effectiveness of their application. Lastly, in our study we used a rack with an Olympic bar that is easily accessible and quick to set up for the deceleration-specific isometric exercises. However, lifting straps were not used during the isometric protocols, which may have limited grip strength and consequently influenced force production in the lower limbs. Future research should investigate the effectiveness of alternative configurations, such as using a belt or harness with chain.

## CONCLUSIONS

Taken together, these findings demonstrate that task-specific isometric pre-activation targeting early deceleration mechanics can effectively enhance COD performance in youth academy soccer players. The DEC_EISO_ protocol produced improvements that exceeded both the SWC and the SRD, suggesting a robust and practically meaningful effect on COD performance. Performance gains peaked approximately 8 minutes post-activation, highlighting the importance of timing in the application of deceleration-specific isometric protocols to acutely enhance COD performance.

## Data Availability

The data supporting the findings of this study are available from the corresponding author upon reasonable request.
